# SARS-CoV-2 Infection and the Newborn

**DOI:** 10.3389/fped.2020.00294

**Published:** 2020-05-22

**Authors:** Fahri Ovalı

**Affiliations:** Division of Neonatology, Department of Pediatrics, Istanbul Medeniyet University, Istanbul, Turkey

**Keywords:** newborn, COVID 19 infection, breast milk, pregnancy, SARS- CoV-2

## Abstract

Severe Acute Respiratory Syndrome Coronavirus Type 2 (SARS-CoV-2) affects people at all ages and it may be encountered in pregnant women and newborns also. The information about its clinical features, laboratory findings and prognosis in children and newborns is scarce. All the reported cases in pregnant women were in the 2nd or 3rd trimester and only 1% of them developed severe disease. Miscarriages are rare. Materno-fetal transmission of the disease is controversial. Definitive diagnosis can be made by a history of contact with a proven case, fever, pneumonia and gastrointestinal disorder and a Polymerase chain reaction (PCR) test of nasopharyngeal swabs. Lymphopenia as well as liver and renal dysfunctions may be seen. Suspected or proven cases of newborns with symptoms should be quarantined in the neonatal intensive care unit for at least 14 days with standart and droplet isolation precautions. Asymptomatic infants may be quaratined at home. Transport of the neonates should be performed in a dedicated transport incubator and ambulance with isolation precautions. There is no specific treatment for the disease, but hemodynamic stabilization of the infant, respiratory management and other daily care are essential. Drugs against cytokine storm syndrome such as corticosteroids or tocilizumab are under investigation. Routine antibiotics are not recommended. No deaths have been reported so far in the neonatal population. Families and healthcare staff should receive pyschological support. Since the infection is quite new and knowledge is constantly accumulating, following developments and continuous updates are crucial.

Coronaviruses are single stranded RNA viruses with a diameter of 60–140 nm and a high rate of genetic mutations and recombinations, rendering them capable of escaping from the immune system and causing novel infections (1). They use the angiotensin-converting enzyme-2 (ACE2) receptors on the cell surface to enter the cell. These receptors are abundant on the surfaces of type II pneumocytes on the lung alveoli, esophageal endothelial cells and gut cells (2). They are highly susceptible to inactivation with heat (56°C for 30 min), 75% ethanol, chlorinated disenfectants or peracetic acid (1). Animals are reservoir for various types of Coronaviruses and humans may become infected through contact with bats, camels and cattle (3, 4). The new SARS-CoV-2 which appeared in Wuhan, China in December 2019 is a beta-Coronavirus which belongs to the same family with the previous Severe Acute Respiratory Syndrome (SARS) virus and Middle East Respiratory Syndrome (MERS) virus (5). The origin of this virus is thought to be the sea-food market in Wuhan but now it spreads easily from human-to-human by aerosols or by close contact. The disease which occurs with SARS-Co-2 is called COVID-19.

Infections with SARS-CoV-2 affects all age groups, and since most children may go unnoticed, they have the potential to spread the virus to other people. It is calculated that a single patient with COVID-19 may infect 2.8 (1.5–6.6) other people (R0), but this rate depends on the attitute of people and on the levels of precautions taken against its spread (6). The incubation period is similar to that of SARS or MERS; i.e., usually between 5 and 6 days but may reach up to 14 days in a small number of cases (7). As of May 8, 2020 there are over 3 950 000 confirmed cases globally, with close to 272 000 fatalities so far (8).

## SARS-Co-2 Infection in Children

Although the virus infects the entire population, infected children were 2% of cases in China, 1.2% of cases in Italy, 0.8% of cases in Spain, 1.7 % of cases in the USA and 1% of cases in Turkey (9–12). Children under 10 years comprised only 1% of all cases. In Korea, 6.3% of all cases that tested positive for SARS-CoV-2−19 were children under 19 years (13). In a report which studied 171 children, 31 (18.1%) were under 1 year, 60.8% were males and 64.9% had pneumonia whereas 41,5% had fever (14). As of April 2, 2020, there were 2572 pediatric cases in the USA. Fifteen percent of these cases (398 cases) occured in children < 1 year. There were 3 deaths among the pediatric cases (11).

The data on the contamination route, susceptibility, clinical findings, pathogenesis, pharmacological treatment and prognosis of the COVID-19 disease in children are limited. The child may acquire the virus through direct contact, as well as through droplets, aerosols or even fecal-oral route (15). Angiotensin-converting enzyme 2 (ACE2) is the main host cell receptor of SARS-CoV-2 and plays a crucial role in the entry of the virus into the cell (16). The expression of ACE2 in the epithelial cells of the lung, intestine, kidney and blood vessels, may explain the high incidence of pneumonia and bronchitis with SARS-CoV-2 (17). SARS-CoV-2 spike protein binding to ACE2 downmodulates ACE2 expression and loss of ACE2 expression results in severe lung damage (18). Estrogens participate in the upregulation of ACE2 expression and this may explain the putative sex predisposition of the virus (19). ACE2 is also protective in acute lung failure (20). Children have generally higher levels of ACE2 than adults and children with confirmed SARS-CoV infection have generally mild symptoms (21). ACE can pass through the placenta, enabling the mother to transfer to baby her immunity and other kinds of protective soluble factors (22). Soluble ACE2 may help children to better counteract the virus. This could help them to contain the virus but also let them to be unrecognized carriers. Circulating levels of ACE2 might have prognostic significance and ACE2 polymorphisms might be a key element of individualized care for its prevention, diagnosis, treatment and monitoring (23). In 97% of cases, symptoms appear by the 10th day (24). Common symptoms include fever, dry cough and fatigue with a few upper respiratory symptoms such as nasal congestion or runny nose and some patients may have gastrointestinal symptoms such as abdominal discomfort, nausea, vomiting, abdominal pain and diarrhea. In 15.8% of children, there were no symptoms or radiologic findings. Lymphopenia was detected only in 3.5% of them (11). Of the 171 infected children, only 3 children with co-morbidities required mechanical ventilation. There were a couple of children with pulmonary findings but were asymptomatic at all (5). Most of the children recover within 1–2 weeks after onset (25).

Most infected children have mild clinical manifestations but they may be contagious. The reason why SARS-CoV-2 infections in children are mild remains elusive. The immune response of newborns to SARS-CoV-2 infections may be qualitatively different with respect to adults. On the other hand, simultaneous presence of other viruses in the lungs and upper airways of children, which is quite common, may limit the growth of SARS-CoV-2 by competition or viral interaction (17). Other reasons include lack of smoking, less exposure to air pollution and fewer underlying chronic conditions in children (12, 26).

Since they are asymptomatic or mildly symptomatic, the majority of children do not undergo diagnostic investigations. Children who become infected with SARS-CoV-1 may have more upper respiratory tract than lower respiratory tract involvement (27). However, extended shedding in nasal secretions and stool may have remarkable implications for community spread in kindergartens, schools and at home (27). Therefore, the role of children in community-based viral transmission should be carefully investigated to understand how much it can actually affect public health (28).

## SARS-CoV-2 Infection in Pregnant Women

Viral pneumonia in pregnant women is generally associated with premature rupture of membranes, preterm labor, intrauterine fetal demise, intrauterine growth restriction and neonatal death (29). Since SARS-CoV-2 is a new virus, there is scarce data on the effects of infection in pregnant women; whether there is any difference with other adult infections, risk of vertical transmission to the fetus and the effects on the fetus, if any.

The majority of pregnant women with COVID-19 disease will have mild or moderate flu-like symptoms. Some women may have fever, cough and shortness of breath. Pneumonia and marked hypoxia are commonly described in older women, who are immunosuppressed or have chronic diseases such as diabetes, cancer and chronic lung disease. However, there can be a group of asymptomatic women or those with minor symptoms carrying the virus; the incidence of such women is unknown (30).

Experts from the World Health Organization (WHO) visited various regions of China between 16 and 24 February 2020 and analyzed 147 pregnant women. Eight percent of these women had severe disease and 1% were in critical condition which led the experts to conclude that the disease did not pose a high risk to pregnant women. Vertical transmission was not analyzed in this study (31). However, Hantoushzadeh et al. have reported 7 pregnant women from Iran, presenting with severe COVID-19 disease, and died in their latter second and third trimester. Three of the 7 women had stillbirth and 6 of their offspring (2 set of twins) died after birth (32). Recently, another infected pregnant woman had miscarriage at the 19th week of gestation. She delivered 4 days after the onset of symptoms. Amniotic fluid and vaginal swabs were negative for SARS-CoV-2, as well as fetal lung, liver and thymus biopsies. However, a placental biopsy obtained immediately after delivery under sterile conditions was positive for SARS-CoV-2. Placental findings demonstrated mixed inflammatory infiltrates in the subchorial space and increased intervillous fibrin deposits. Funisitis was also present. Any bacterial and fungal infections were ruled out (33).

Medical treatment of pregnant women is controversial. Hydroxychloroquine has been used widely in pregnant women for the treatment of lupus erytematosus or malaria, without any significant side effects (34). Remdesivir is a nucleotide analog which is active against all coronaviruses including SARS, MERS and SARS-CoV-2. Phase 3 trials for its efficacy in SARS-CoV-2 positive pregnant women are under way (ClinicalTrials.gov number NCT04280705, NCT04252664, and NCT04257656). Lopinavir/ritonavir has proven to be a safe drug in HIV patients without any increased risk of fetal anomalies, preterm birth or low birthweight infants; it may be used in pregnant women, if deemed necessary (35).

## Materno-Fetal Vertical Transmission

Chinese National Health Commission issued a statement on 8 February 2020, which recommended that the pregnant women should be cared carefully, infection control measures should be taken at the delivery clinics including wearing PPE; and isolation of suspected or proven cases of COVID-19 for 14 days. They also suggested to stop breastfeeding the infant and prevent close contact of the mother with the infant (36). However, it must be underlined that this statement is not evidence-based, but rather reflects the opinions of an expert committee.

Transmission of SARS-CoV-2 from the infected pregnant woman to the fetus is still controversial. Viremia is seen in only 1% of COVID-19 cases, suggesting that placental and fetal seeding might be quite rare (37). However, if viremia is present, the disease is more severe (38). The ACE2 receptor is widely expressed in the placenta, with a similar receptor binding domain structure of SARS-CoV-2. However, until now, Polymerase Chain Reaction (PCR) findings of suspected fetuses have been negative, as well as negative amniotic fluid and placenta findings (39).

Zeng H et al. reported 6 infected mothers and their infants. Although PCR results were negative both in mothers and infants, 2 infants had elevated specific IgM and IgG levels, suggestive of an intrauterine infection (40). Three other infants with pneumonia on the 2nd day of life were reported by Zeng L. et al. (41). Although they were delivered by cesarean section under infection control procedures, they tested positive for SARS-CoV-2 on nasopharyngeal and anal cultures. It is possible that early infection might have occured by postnatal early contact with the infected mother. In another report by Dong et al. a 37 week girl, born to a SARS-CoV-2 positive mother in a negative pressure room, after taking all precautions, had high specific IgM levels at 2 h of age. Cytokines and white blood cell counts were also elevated. Her PCR results were negative for 5 consecutive swabs during the first 16 days of life. Although decreased significantly, her IgM and IgG levels were still elevated at the 16th day. Since IgM antibodies are elevated only after 3–7 days after the infection, high IgM levels in the infant only at the 2nd h of birth strongly suggested an intrauterine infection. PCR testing of the amniotic fluid and placenta were not performed in this infant (42). Kimberlein and Stagno argued against this finding, stating that “most congenital infections are not diagnosed based on IgM detection, because IgM assays can be prone to false positive and false negative results, along with cross reactivity and testing challenges.” (43). The sensitivity and specifity of IgM assays which are 70.2 and 96.2%, respectively, are lower than those of PCR testing (44). (Table 1) IgM testing in congenital cytomegalovirus, toxoplasma, syphilis and Zikavirus infections is not alone sufficient enough for definitive diagnosis of the relevant diseases. Furthermore, the rapid decay of IgM levels within 14 days lend support to the reasoning that high IgM levels might not represent a true infection.

**Table 1:** Reports on infants with SARS-CoV-2 infection.

**Author**	**Infant**	**Delivery route**	**Clinical findings**	**Outcome**
Zeng et al. (45)	5 days male	C/S^*^	Fever, pneumonia	Discharged well
	2 days male	C/S	Fever, lethargy, vomiting, lymphopenia, pneumonia	Discharged well
	2 days male	C/S	31 wks preterm, respiratory symptoms, bacterial sepsis, NIV^**^	Discharged well
Dong (46)	2 h female	C/S	Asymptomatic infant, IgM(+)	Discharged well
Zeng et al. (47)	17 days, male	V^***^	Fever, cough, runny nose, vomiting	Recovered well
Zhang et al. (48)	30 h male	C/S	Shortness of breath	Discharged well
	5 days male	C/S	Fever	Discharged well
	5 days female	C/S	Asymptomatic	Discharged well
	17 days male	C/S	Fever, cough, vomiting	Discharged well
Aghdam et al. (49)	15 days male	C/S	Fever, mottling, tachycardia, mild retraction	Discharged well
Wang et al. (50)	36 h male	C/S	Asymptomatic	Clinically well
Buonsenso et al. (51)	15 days male	C/S	Asymptomatic	Clinically well
Sinelli et al. (52)	5 days male	V	PCR (+) at 48 h, respiratory distress on day 5, chest x-ray: mild ground-glass opacities	Discharged well

* *C/S: Cesarean Section*.

** *NIV: Non-invasive ventilation*.

*** *V: Vaginal delivery*.

On the other hand, there are some data that SARS-CoV-2 can be transmitted through the fecal-oral route (15). Transmission of the virus is possible during vaginal delivery, by direct contamination of the infant by vaginal secretions, as well as through the droplets of the infected mother in the immediate postpartum period, if no PPE is used.

It was not possible to prove the vertical transmission of the SARS virus during the SARS epidemic (53). Since SARS-CoV-2 shows 85% homology with the original SARS virus, it may be assumed that the new virus behaves similarly. However, although PCR remains as the gold standard for the diagnosis, clinical findings and chest computed tomography (CT) findings should be investigated thoroughly (44). Since all the pregnant women who had been infected were in their 2nd or 3rd trimester at the time infection, it is impossible to have an idea on the transmission dynamics of the infection during the whole pregnancy. It is well-known that the rate of transmission of rubella infection during pregnancy is higher in the first or second trimester, but not in the third trimester (54). On the other hand, fever is a common manifestation of SARS-CoV-2 infection and high temperature may be a theoretical concern during the organogenesis period in the first trimester and associated with increased risk of congenital anomalies or miscarriage. Therefore, more information on the transmission rates of the SARS-CoV-2 during the first and second trimester will be available after some months; i.e., when the women who got pregnant during the pandemic delivered 9 months later.

## Optimal Delivery in Infected Mothers

There is still a controversy on the optimal delivery method of infected mothers (9, 55, 56). In pregnant women with SARS-CoV-2 infection, there is not any indication for routine cesarean delivery except for obstetrical reasons. However, in many case reports, cesarean delivery was preferred most of the time, aiming for reducing hospital stay of mothers, minimizing chance of cross-infection, reducing maternal physical exertion during delivery and ensuring safety of other people at the clinic (57). Iqbal et al. have reported a 39 week infant, born through vaginal route, and discharged home on the 6th day without any complications (58). In a systematic review of 8 studies, comprising 100 women, 85% of them had cesarean section, 29% had delivered a premature infant and 16% of the infants were low birthweight (59). If cesarean section is preferred, it should be performed by a senior physician in order to minimize possible complications. Since general anesthesia is considered as an aerosolizing procedure, personal protective equipment (PPE) including N95 masks, long-sleeved scrub or jumpsuit, goggles, face shields, two-layer gloves, should be worn by all staff during the operation. Alternatively, epidural anesthesia may be preferred.

Although there is limited data, rupture of membranes does not pose an additional risk (59). However, since feces might contain virus, caution should be taken, especially during vaginal birth (45).

Delivery is not indicated in a pregnant women with non-severe illness. Preterm delivery should be considered only by obstetric reasons. However, severely ill patients at least 32–34 weeks of gestation with SARS-CoV-2 pneumonia or patients who do not improve by treatment may benefit from early delivery even in the absence of obstetric indications (46, 57). In critical cases, there is some evidence that early delivery may improve maternal oxygenation, regardless of gestational age (60).

Antenatal corticosteroids for the prevention of respiratory distress syndrome and other morbidities in the preterm infant is not contraindicated in women with confirmed SARS-CoV-2 infection, although it is known that they accelerate the development of type 2 alveolar cells which are rich in angiotensin converting enzyme 2, a co-receptor for SARS-CoV to enter the cell (45). Moreover, antenatal corticosteroids have been used safely in pregnant women with influenza and HIV infection (61).

## Delivery Room Management

Delivery should take place in a room with negative pressure and all staff should wear PPE including the pediatrician attending to the delivery. If a room with negative pressure is not available, a separate room should be used. The number of staff attending to the delivery should be kept at the minimum. If needed, neonatal resuscitation is performed according to the Neonatal Resuscitation Program guidelines by an experienced person with PPE. Initial care of the newborn should not be delayed due to COVID-19, and should be done according to standard procedures. If clinically stable, the newborn should be bathed after birth in order to remove virus potentially present on the skin (62).

Some experts advocate refraining from delayed cord clamping but this recommendation is not evidence-based and American College of Obstetrics and Gynecology (ACOG) recommends no change in the practice of delayed cord clamping in COVID-19 cases until there is sufficient evidence (46).

Obstetric and neonatology clinics should work in close collaboration for the management of these cases and the neonatology team should be notified at least 30 min before delivery in order to make necessary preparations.

## SARS-CoV-2 Infection in the Newborns

Case reports or randomized controlled trials on SARS-CoV-2 infection in the neonates are limited and are summarized in Table 1.

The first newborn in the World, infected with SARS-CoV-2 was a 17 days old boy with fever, cough, runny nose and vomiting. He recovered with symptomatic treatment (47). Chen et al. reported 9 infants of positive mothers, all delivered by cesarean section. Four of them were premature. None of the infants was positive for SARS-CoV-2, including amniotic fluid, umbilical cord blood, nasopharyngeal swab and breast milk (39). Liu et al. reported 3 infected women; their infants tested negative for SARS-CoV-2, and all of them were clinically well (45). Zhu et al. reported 10 infants from 9 mothers (1 twins). Seven mothers had delivered by cesarean section whereas 2 mothers had had vaginal birth. Although some of these infants were symptomatic with respiratory symptoms, tachycardia, feeding intolerance and fever, none of them tested positive for SARS-CoV-2. In this group one preterm infant died at the 9th day of life with multiple organ dysfunction, disseminated intravascular coagulation and shock (63). Wang et al. reported an infant born at 30 weeks of gestational age with cesarean section to an infected mother. The infant was negative for SARS-CoV-2 both on the first day and on the 9th day (64). Zhang et al. reported 4 newborn infants between 30 h and 17 days. All of the mothers were infected with SARS-CoV, showing symtoms before and 1 week after delivery. Cesarean section was used for all of them. Two of the infants had fever, 1 had shortness of breath, 1 had cough and 1 had no symptoms. None of them required intensive care and all of them were discharged well. Three of them were separated from their mothers and were not breastfed (48). Another infant, who had had close contact with infected relatives presented at 55th day of life with cough and runny nose and bilateral ground glass apperance on the lungs. There were slight elevations of liver function tests, myocardial enzymes; CD8 T-lymphocytes and serum IgM levels. She was isolated and started on empirical antibiotics as well as inhaled interferon α-1b (15 μg, bid), reduced glutathion, ursodeoxycholic acid and traditional Chinese medicine lotus qingwen. A feces sample at the 11th day proved positive for SARS-CoV-2 (65). Chen Y et al. reported 4 infants, 3 delivered by cesarean section and one by vaginal route. The infants tested negative for SARS-CoV-2. They were separated from the mother after birth and fed with formula. They were discharged well (66). In another case-control study, Li et al. reported 17 newborn infants born to SARS-CoV-2 positive mothers. Preterm birth rate was 23.5%, and low birthweight rate was 17.6%; both were higher than those of normal population, but was attributed to pregnancy complications rather than COVID-19 itself (67). In another case report, Aghdam et al. reported a 15-day old newborn who presented with fever and mottling, accompanied by tachycardia, tachypnea and mild subcostal retraction without cough, desaturation, runny nose or gastrointestinal symptoms. He was discharged 6 days later in good health (49). Wang S et al. reported an asymptomatic male infant diagnosed at 36 h after birth (50). Recently, Buonsenso et al. reported 2 newborns born to mothers with COVID-19 in pregnancy. The first one was delivered by cesarean section at 38 weeks. He tested negative on day 1 and day 5 of life, but became positive at day 15, although he was clinically well. The mother was breastfeeding the infant. Milk samples tested negative and respiratory secretions were the probable source of the infection. Maternal immunglobulin G and breastmilk antibodies might have protected the newborn from symptomatic infection. The second newborn was delivered by cesarean section at 35 weeks and he was asymptomatic with negative tests on day 1, 5, and 18. The father was feeding the baby with expressed breastmilk (51). Sinelli et al. reported a baby born to a mother with COVID-19. On the second day after vaginal delivery, the infant tested positive for SARS-CoV-2. The mother and the infant were not separated, but isolated in the same room. 48 h after isolation, the infant developed cyanosis, respiratory distress and poor sucking. He was placed on 30% oxygen and high flow nasal cannula. Chest radiograph showed mild bilateral ground-glass opacities. Respiratory support was discontinued 50 h after NICU admission and he was discharged in good condition on day 18 of life (52).

### Clinical Manifestations in the Newborns

Neonatal infection with SARS-CoV-2 may begin insidiously. The most prominent characteristic of the infection in young children is a history of contact with a proven case of COVID-19 (commonly the mother) or travel history to an epicenter. Diagnosis is confirmed by the demonstration of nucleic acids of the virus by real time PCR in the respiratory tract swabs (36).

There is no clinical finding specific to the newborns. The body temperature may be high, normal or low. S/he may have respiratory symptoms such as cough, tachypnea, apnea, grunting, nasal flaring, and tachycardia as well as lethargy, vomiting, diarrhea and abdominal distention (68, 69). In severe cases and in cases with immune deficiency, congenital heart disease, bronchopulmonary dysplasia, respiratory tract anomalies, severe malnutrition or anemia, the findings should be interpreted more cautiously.

### Laboratory Findings

There are no specific laboratory findings. White blood cells may be normal or elevated and lymphocytes may be decreased. Mild thrombocytopenia, mild elevations of creatine kinase, alkaline phosphatase, alanine aminotransferase, aspartate aminotransferase and lactate dehydrogenase may be seen. The virus may be isolated from the upper respiratory tract, endotrachaeal aspirate, blood or feces. Pneumonic infiltrations may appear on chest radiography, lung ultrasonography or computed tomography (CT) of the lung (70).

### Suspected Newborns

All newborns born to a mother with confirmed COVID-19 within 14 days before birth or 28 days after birth, or who had had direct contact with any person with confirmed infection are accepted as suspected cases. All suspected newborns should be quarantined (70). This does not mean that all suspected newborns should be hospitalized; if they are clinically stable, they can be managed at home. Neonatal Intensive Care Unit (NICU) admission should be reserved for neonatal clinical reasons or when close monitoring is indicated.

In suspected cases, PCR testing should be done first at about 24 h of age. Repeat testing should be done about 48 h of age. For well-newborns who will be discharged prior to 48 h, this test may be omitted. However, in rare cases, the infant may test negative in 24 h but positive in 48–72 h. Additional testing may be considered for sick infants requiring prolonged hospial care (71).

### Confirmed Newborns

If any of the following is positive, the case is regarded as positive (36):

- Positive PCR for SARS-CoV-2 in respiratory tract or blood samples
- High homology of viral gene sequences of the samples from the respiratory tract or blood to the COVID-19 sequence.

### Management of Asymptomatic Newborns

Whether suspected or confirmed, asymptomatic infants should have a complete blood count, C-Reactive Protein (CRP) and Real Time-PCR for SARS-CoV-2. It is preferable to take the samples at least from 2 sites, including the upper respiratory tract, lower respiratory tract or blood. Feces may be obtained and kept for further analysis (72). The infants should be kept in an isolated room at least for 14 days. If deemed stable, the infant may be discharged home, provided that s/he is kept in an isolated room for 14 days. If the infant was kept with other newborns in the same room previously, these infants should have an SARS-CoV-2 test immediately and kept in quarantine until the tests are negative (73). Feeding of the infant depends on the infectious status of the mother. Recent evidence suggests that even non-symptomatic individuals may spread COVID-19 and conventional measures of protection such as face masks provide insufficient protection. Therefore, healthcare staff should wear PPE while dealing with suspected cases (74).

### Management of Symptomatic Newborns

These infants require the above mentioned laboratory tests and an additional chest radiography and/or chest CT. Liver and kidney function tests and cardiac enzymes may be required. Since young infants may have other respiratory tract viral infections, viral pathogens may be sought. They should be quarantined and closely monitored until the results are negative. Feeding depends on the infectious status of the mother. If RT-PCR for SARS-CoV-2 is negative, the infant should be treated according to the other possible disease (69). The healthcare staff should wear PPE.

Infected infants may be discharged from the hospital if

- There is no fever for 3 consecutive days
- Respiratory symptoms resolve
- Severe lung radiological findings resolve
- Nasopharyngeal swabs taken 24 h apart are negative (62).

### Neonatal Transport

Newborns should be transported within the hospital and between the hospitals in a closed, dedicated incubator and ambulance. The ambulance should be equipped with a ventilator and necessary drugs, surface disinfectants and hand disinfectants. Before and after the transport, the incubator and the ambulance should be disinfected. The transport personnel should wear a PPE (70).

## Neonatal Intensive Care Unit Design and Procedures

The NICU may be designed in 2 sections: a quarantine section and a non-infectious section. The attending neonatologist should decide on which baby should be admitted to which section. An algorithm for this purpose will be helpful. Suspected or confirmed cases should be kept in the NICU, in separate rooms, in closed incubators. The decision on the separation of the mother and infant depends on the clinical condition of the mother and infant, the physical configuration and infrastructure of the unit, the test results and the will of the mother to breastfeed her baby. Instruments such as stethescopes, thermometers, laryngoscopes etc. should be private for each patient. If the neonatal unit is very busy, infants with similar findings may be cohorted in the same ward, all 2 meters apart from each other (70).

All involved staff in the NICU should conform to the precautions which include but not limited to wearing hospital scrubs, shaving facial hair, taking off accessories such as watches, bracelets, rings, keeping nails short and long hair tied, wearing dedicated shoes at the hospital, wiping cell phones and other personal accessories, notwithstanding putting on PPE (70, 71). It is noteworthy to state that COVID-19 disease may be spread by non-symptomatic persons and is considered as Group-A infection, a category for highly infectious pathogens, such as cholera and plague (75). The order of donning and doffing of PPE should be observed by every personnel (76). Aerosolization should be minimized and during invasive procedures to the respiratory tract (endotracheal intubation, bagging, aspiration, bronchoscopy or laboratory sampling, nasal cannula oxygen flow > 2 Liter/min, or mechanical ventilation), additional water-resistant gowns, N95 masks, head shields and feet shields should be used. Intubation by the videolaryngoscopic technique may reduce exposition to airborne particles. After the insertion of the endotracheal tube, the tube may be clamped, connected to the ventilator and the clamp is removed in order to to avoid aerosol and droplet spread (clamped intubation technique) (77). The door of the room should be kept closed at all times and entrance to the room should be restricted to the minimum number of people. The equipment used for the patient should not be used concomitantly for another patient. Visitation to the unit should be restricted. For parents or father, a camera may be helpful. The air circulation in the unit should be increased. Medical waste of infected patients should be disposed of separately in double layer boxes. The linens and other textile should be treated with a chloride solution at least for 10 min and then washed at 60–90°C, separately. The room of the patient should be disinfected thoroughly after discharge (78).

## Treatment

Treatment is mainly symptomatic. Supportive treatment including fluid-electrolyte treatment, maintaining hemodynamic stability of the infant, parenteral or enteral nutrition as well as respiratory support are essential. Conventional mechanical ventilation, high frequency ventilation or nitric oxide therapies may be tried. In critical cases, continuous renal replacement therapy or extracorporeal membrane oxygenation (ECMO) may be helpful.

Anti-viral treatment is not generally needed in newborns and there is no data on the efficacy of anti-viral drugs in the newborn population (70). The recommendation by Zhejiang University School of Medicine comprises the use of nebulized interferon alpha-2b and oral lopinavir/ritonavir (79). In older children and in children with severe pulmonary findings, hydroxychloroquine, azithromycin and lopinavir+ritonavir may be used. Hydroxychloroquine is an anti-malarial drug, which is used in autoimmune diseases also. It increases the endosomal pH, inhibiting virus-cell fusion. It also inhibits the entry of SARS-CoV into the cells and interferes with glycosylation of cellular receptors of SARS-CoV. Furthermore, it may have an immune-modulating effect (80). Mechanism of action implies that hydroxychloroquine needs to be given at the beginning of the infection. The possibility of drug toxicity including QT prolongation and retinal toxicity especially in individuals with epilepsy, porphyria, myasthenia gravis and glucose-6-phosphate dehydrogenase (G6PD) deficiency should be considered (81). Remdesivir is a nucleotide analog and acts on viral RNA transcription after entering the cell by inhibiting RNA polymerase and seems to be safe and effective in the adult population (82).

Cytokine storm syndrome, which appears at the final stage of the disease is frequently related to extensive tissue damage with lung involvement and multi-organ failure. The protagonist of this storm is interleukin-6 (IL-6). In order to antagonize hyperinflammation, IL-6 blockade or immunosupression with corticosteroids can be hypothesized. Veronese et al. have conducted a meta-analysis on the use of corticosteroids in COVID-19 patients (83). Four studies and 542 patients were included in this meta-analysis. Two studies reported negative findings regarding the use of corticosteroids in COVID-19 patients, one study did not report any benefit but one study which included 201 participants with different stages of COVID-19 pneumonia found that in severe forms, the administration of standard doses of methylprednisolone significantly reduced the risk of death by 62% (84). Available literature does not fully encourage the routine use of corticosteroids in COVID-19 (82). There is one ongoing trial, pending results (85). Tocilizumab is recombinant humanized monoclonal antibody which binds to IL-6 seceptor and blocks its function (86).

On the other hand, since ACE2 receptors play an important role in the development of the disease, recombinant ACE2 may be a treatment option for patients with severe COVID-19 (ClinicalTrials.gov NCT04287686) Lopinavir-ritonavir appears to have little role in the treatment of COVID-19 disease (87). Drugs like remdesivir or lopinavir-ritonavir can be considered as compassionate treatment, after careful consideration of the risk-benefit ratio and technical issues (88). Other treatment options such as convalescent plasma and anakinra are under investigation (89, 90).

Antibiotics may be used if there is secondary bacterial infection. Standard immunglobulins or hormonal treatments are not helpful. We do not have any information on the long-term effects of COVID-19 acquired in the neonatal period. No deaths have been reported so far among neonates.

## Post-Discharge Care

If the infant tests positive without any symptoms, s/he can be sent home, but should be followed up by outpatient visits, telemedicine or telephone calls for 14 days. All caretakers at home should have hand hygiene, masks, and gloves. Uninfected persons older than 60 years of age or with co-morbid diseases should not provide care for these infants (91). If the infant is negative but the mother is positive, an uninfected person should take care of the infant. These caretakers as well as the mother should stay at least 2 meters away from the infant and should use a mask and practice hand hygiene when getting into contact with the infant. In adults, viral shedding has been reported after nasopharyngeal swabs become negative, because viral replication and clearance are decided by the body defense mechanisms. How long an infant sheds the virus is currently unknown, but may be as long as 22 days (92).

## Breastmilk

Breastmilk is generally considered safe against viral infections because of its protective contents such as immunoglobulins and other bioactive compounds. Breast milk may contain anti- SARS-CoV-2 antibodies in infected mothers but there is no data yet on this issue. On the other hand, various case reports have concluded that breast milk does not contain the virus RNA (93, 94). If the PCR test is negative, the infant may be breastfed safely. However, if the mother tests positive, the recommendations for breastfeeding becomes controversial. There is no data supporting the notion that these infants should not be breastfed. Academy of Breastfeeding Medicine recommends breastfeeding after taking all possible precautions (93). WHO recommends breastfeeding, after taking necessary precautions. The mothers should be encouraged for breastfeeding. They should put on appropriate PPE, wash her hands before and after breastfeeding, and wash clothes at 60°C, after the contact (95). However, since the infant and the mother are together, the possibility of airborne transmission can not be ruled out. The mother should not hug or kiss the infant. If the mother and infant is going to stay together, there should be at least 2 meters between the beds. It should not be forgotten that with this practice, a person without any PPE (i.e., the infant) gets into close contact without any social distancing (i.e., sucks the breast) with another person with suspected or confirmed infection (i.e., the mother). This type of contact is not allowed in adults, but it may be allowed between the mother and her child. Centers for Disease Control (CDC) recommends that, if the mother has suspected or confirmed COVID-19 infection, the option of separate management of the mother and child should be considered as the first choice and the risks and benefits of this separation and consequences of not starting, continuing or suspending breastfeeding should be shared with the family and documented (96). Guidelines issued by the Italian Society of Neonatology and endorsed by the Union of European Neonatal and Perinatal Societies (UENPS) suggest that if a mother who is SARS-CoV-2 positive or is a person under investigation, or who is asymptomatic or has few symptoms at delivery, rooming in is feasible and direct breastfeeding is advisable but with strict infection control measures. If however, the mother is too sick to care for the newborn, the neonate will be managed separately and fed fresh expressed breast milk, with no need to pasteruize it, as human milk is not believed to be a vehicle of SARS-CoV-2 (97). Chinese Pediatrics COVID-19 WorkingGroup also advocates formula or donor breast milk, albeit without evidence (98). Therefore, the risks and benefits of breastfeeding should be balanced. If close contact is not preferred, expressed breast milk may be preferred and given to the infant by an uninfected caregiver. This practice may have some drawbacks also; such as preventing the bonding between the infant and the mother. The benefits of breastfeeding outweigh any risk of transmission of the virus through the breastmilk. This guidance may change as knowledge evolves.

Hydroxychloroquine is considered safe if used during lactation but nothing is known on the passage of remdesivir to human milk (99).

## Psychological Support

Rising fears about the infection should be reduced and sound knowledge should be shared by the family. The information in the social media should be interpreted cautiously. Education and shared decision making empowers the family. If the infant is separated from the mother, the mother and the whole family may suffer from anxiety and stress. Support from a psychologist or a social worker may be sought. On the other hand, health staff working with COVID-19 cases may develop various pyschological manifestations due to heavy work load, shortage of equipment and guarded prognosis of the patients. Therefore, they may also need pyschological support.

## Conclusion

SARS-CoV-2 infection is a new disease with many unknown issues. With emerging evidence, pathophysiology and management options change. The knowledge on vertical transmission of the disease and on clinical manifestations in the newborns is expected to accumulate in the forseeable future. There is a growing body of evidence on the subject and continuous updates are important to implement current knowledge in the management of COVID-19 in infants and children.

## Author Contributions

The author confirms being the sole contributor of this work and has approved it for publication.

## Conflict of Interest

The author declares that the research was conducted in the absence of any commercial or financial relationships that could be construed as a potential conflict of interest.
